# Synthesis of Optimized Molecularly Imprinted Polymers for the Isolation and Detection of Antidepressants via HPLC †

**DOI:** 10.3390/biomimetics4010018

**Published:** 2019-02-20

**Authors:** Alexander D. Hudson, Ricard Solà, Jorge T. Ueta, William Battell, Oliver Jamieson, Thomas Dunbar, Beatriz Maciá, Marloes Peeters

**Affiliations:** 1 Manchester Metropolitan University, Faculty of Science and Engineering, Division of Chemistry and Environmental Science, Manchester M1 5GD, UK; rsolamestres@gmail.com (R.S.); oliver.jamieson@stu.mmu.ac.uk (O.J.); thomasdunbar1993@gmail.com (T.D.); B.Macia-Ruiz@mmu.ac.uk (B.M.); m.peeters@mmu.ac.uk (M.P.); 2 Departamento de Engenharia Química, Escola Politécnica, Universidade de São Paulo, Avenida Prof. Luciano Gualberto, Travessa 3, 380, CEP05508-900 São Paulo, Brazil; tadinchnails@gmail.com; 3 Department of Chemical Engineering, Centre for Advanced Separations Engineering, University of Bath, Claverton Down, Bath BA2 7AY, UK; W.Battell@bath.ac.uk

**Keywords:** molecularly imprinted polymers, fluoxetine, selective serotonin reuptake inhibitors (SSRIs), optical batch rebinding, high-performance liquid chromatography

## Abstract

Antidepressants such as amitryptiline and fluoxetine are on the list of modern essential medicines of the World Health Organization. However, there are growing concerns regarding the ecological impact of these pharmaceuticals, leading to a great need to improve current wastewater treatment procedures. In this contribution, we will report on the use of molecularly imprinted polymers (MIPs) for the extraction of antidepressants in water samples. MIPs were developed for fluoxetine and duloxetine, antidepressants belonging to the class of selective serotonin reuptake inhibitors (SSRIs). The binding capacity of these microparticles was evaluated using ultraviolet–visible (UV–Vis) spectroscopy. A new high-performance liquid chromatography (HPLC) procedure coupled to UV detection was developed, which enabled the study of mixtures of fluoxetine and duloxetine with other nitrogen-containing compounds. These results indicate that it is possible to selectively extract SSRIs from complex samples. Therefore, these versatile polymers are a promising analytical tool for the clean-up of water samples, which will benefit aquatic life and reduce the ecological impact of pharmaceuticals.

## 1. Introduction

**Figure 1:**
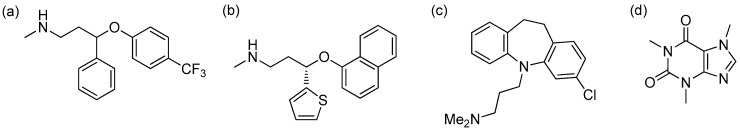
Chemical structure of (**a**) (±)-fluoxetine, (**b**) (*S*)-duloxetine, (**c**) clomipramine, and (**d**) caffeine.

Prescriptions of antidepressants are at an all-time high; in the last decade, the number of antidepressants that have been dispensed by pharmacies in the United Kingdom have increased by more than 100% [1]. Prozac is a common antidepressant that falls under the category of selective serotonin reuptake inhibitors (SSRIs). Fluoxetine (Figure 1), the active component of Prozac, inhibits the reuptake of serotonin in presynaptic neurons leading to an increased availability of serotonin [2]. Selective serotonin reuptake inhibitors are known to ease the symptoms of moderate to severe depression [3] and are used to complement treatment for a variety of disorders, such as obsessive-compulsive disorder [4], bulimia nervosa [5], and panic disorders [6]. The World Health Organization has listed Prozac as one of the essential medicines in modern healthcare, demonstrating the importance of this drug [7]. 

With their increased use and availability, the environmental impact of these antidepressants becomes a greater concern. Pharmaceuticals are widely found in aquatic systems, which is due to a combination of low removal efficiency in sewage treatment plants (STPs) or a lack of available STPs [8]. The presence of SSRIs can have a significant impact on the serotonin levels of fish and other aquatic life, which plays a pivotal role in activity, aggression, and reproductive behavior [9,10]. Average surface water concentrations of fluoxetine can range from ≈0.01 to 1.4 µg/L depending on location [9]. Chu and Metcalfe [11] have demonstrated the bioaccumulation of SSRIs in fish, detecting the presence of fluoxetine and its active metabolite, norfluoxetine, in fish tissue. This has been confirmed by Brooks et al. [12], showing that the level of fluoxetine in organs in fish is similar to levels in the environment. 

Liquid chromatography–mass spectrometry (LC–MS) [13,14], gas chromatography–mass spectrometry (GC–MS) [15], and nuclear magnetic resonance (NMR) spectrometry [16] are commonly used techniques for fluoxetine measurements, but these are costly, time-consuming, and frequently involve cumbersome procedures for sample preparation. High-performance liquid chromatography (HPLC) coupled to either ultraviolet–visible (UV–Vis) or fluorescent detection has the advantages of high selectivity and high sample throughput [17]. Here, we explore the use of molecularly imprinted polymers (MIPs) as sorbents due to their low-cost, high recovery, short removal time, and ability to perform extraction in aqueous samples. Molecularly imprinted polymers are porous polymers containing binding sites with high affinity for their target molecule [18,19]. These polymers can be easily tailored for multiple targets, which allows for the simultaneous extraction of fluoxetine as well as a range of other compounds within the same family of antidepressants. 

The reports on MIPs for fluoxetine detection in literature are sparse. Nezhadali et al. [20] reported the use of pyrrole as a functional monomer along with spectrophotometric determination to selectively detect fluoxetine. Barati et al. [21] synthesized MIPs as solid phase extraction (SPE) sorbents through coprecipitation, employing magnetic chitosan and graphene oxide as monomers. A range of biological and environmental samples was studied; however, the main drawback with this procedure is the time-consuming and complicated synthesis of the hybrid MIP structures, which is not scalable. Molecularly imprinted polymers for the detection of fluoxetine can also be combined with electrochemical sensing, as demonstrated by Alizadeh et al [22]. 

For environmental purposes, it is preferable to detect a range of SSRIs instead of focusing solely on fluoxetine. We demonstrate that when imprinting fluoxetine as the template, we can develop materials that are suitable sorbents for fluoxetine but also other SSRIs, such as duloxetine (Figure 1). We monitor the extraction of a range of SSRIs with HPLC coupled to a UV–Vis detector. A range of monomers was evaluated, showing the design of the polymers was crucial to tuning the specificity and selectivity of the synthetic recognition elements. Similar levels of binding were achieved for all compounds within the fluoxetine family, indicating that these MIPs are powerful sorbents to be used during the pre-treatment of drug samples for analysis with HPLC. In the future, these materials are likely to have high potential for the treatment and clean-up of water samples and may be used in wastewater facilities.

## 2. Materials and Methods

### 2.1. Reagents 

All solutions were prepared with deionized water or phosphate-buffered saline (PBS) solutions. Phosphate-buffered saline solutions were prepared using Dulbecco tablets obtained from Oxoid Limited (Basingstoke, UK). (±)-Fluoxetine hydrochloride salt (99%), and (*S*)-duloxetine hydrochloride salt (99%) were both purchased from TCI Chemicals Ltd. (Oxford, UK). Clomipramine hydrochloride salt (98%), initiator azobisisobutyronitrile (AIBN) and caffeine were purchased from Sigma-Aldrich (Gillingham, UK). Methacrylic acid (MAA), ethylene glycol dimethacrylate (EGDMA), 2-(hydroxyethyl)methacrylate (HEMA), acrylamide (AA), itaconic acid (IA), acrylonitrile, 2-vinylpyridine (2-VP), toluene, methanol, and dimethylsulfoxide (DMSO) were purchased from Acros (Loughborough, UK). Prior to polymerization, the stabilizers in all monomers and crosslinker monomers were removed by passing the solutions over a column packed with alumina (Sigma-Aldrich). Solutions of HCl and NaOH (0.1 mM) in water were used to adjust the pH values of these solutions. 

### 2.2. Molecularly Imprinted Polymer Synthesis and Optimisation

(±)-Fluoxetine hydrochloride (0.16 mmol) was dissolved in DMSO (7 mL) together with a functional monomer (0.32 mmol). The functional monomers that were used for the synthesis of fluoxetine MIPs (MIP-1-7) are described in Table 1. Subsequently, the crosslinker monomer (EGDMA, 0.96 mmol) was added followed by the addition of the initiator (AIBN, 10 mg, 0.06 mmol). The solution was bubbled with N_2_ for five minutes, then heated to 65 °C to initiate the polymerization. The reaction was kept at constant temperature for 16 h, ensuring the polymerization reached completion. Next, the resulting polymer was ground into a fine powder using a planetary ball mill (model PM100, Retsch Ltd., Hope, UK), using a 50 mL stainless steel grinding jar and five stainless steel grinding balls of 1 cm diameter each. Three Soxhlet extractions were performed sequentially on the grounded polymer, with different solvent mixtures: (i) methanol/deionized water (50:50); (ii) acetonitrile/glacial acetic acid (50:50); and (iii) methanol/deionized water (50:50). The extraction was performed until no traces of the template were observed in the filtrate using UV–Vis spectroscopy. Thermogravimetric analysis (TGA) with a TG4000 from Perkin Elmer (London, UK) was performed to assess the stability of the polymer after extraction of the template. The particles were dried under vacuum for 16 h, reground, and sieved to obtain only particles with sizes smaller than <25 µm. Nonimprinted polymers (NIP-1–7) were prepared in the same manner but without the addition of the template during the polymerization. MIP-8 was also prepared using the same protocol and IA as monomer, but using a 1:1 mixture of fluoxetine and duloxetine as a template instead of just fluoxetine. To compare the specificity of the polymers towards fluoxetine, imprint factors (IFs) were determined by fitting the data with an allometric fit for concentrations of 0.05 and 0.25 mM. 

### 2.3. Batch Rebinding Experiments Evaluated by UV–Vis Analysis

Optical batch rebinding experiments were evaluated by measuring absorbance values (*λ*_max_ = 264 nm) of the solutions with an Agilent 8453 spectrophotometer (Stockport, UK). For each experiment, MIP or NIP powder (10 mg) was added to several solutions of antidepressants in PBS (5 mL) with different known concentrations. The resulting suspensions were placed on a STUART SSM1 orbital shaker (Cole-Palmer, Stone, UK) at 125 rpm for 90 min and passed through 0.45 µm filters prior to UV–Vis analysis. 

Various fluoxetine concentrations, ranging from 0 to 1.5 mM in PBS, were used to construct dose-response curves for MIP-1–7. These curves were then employed to determine the free concentration of the template in the filtered solutions (*C_f_*) by dividing the absorbance over the gradient of the calibration curve. Subsequently, the amount bound to template (*C_b_*) was calculated by subtracting the initial concentration added to the solution (*C_i_*) by the free template concentration of the filtered solution (*C_f_*). The moles of template bound per gram of polymer (*S_b_*) was obtained by dividing *C_b_* × *V*, where *V* is the volume in liters, over the amount of polymer added in the rebinding experiment (10 mg). The IF was determined by dividing the *S_b_* for a given MIP by the *S_b_* for the corresponding NIP.

As a measure of specificity and in order to compare the different MIP compositions, the IF was determined at a free concentration of the template (*Cf*) of 0.05 and 0.2 mM. This was done by fitting the MIP data with an allometric fit (*y* = *ax^b^*) which is in line with the heterogeneity of the binding sites. The data generated with the NIP, in general, followed a linear fit line, which is to be expected for nonspecific binding. The amount of analyte bound at *Cf* was determined according to the fit for both MIPs and NIPs, with an IF calculated at a given concentration. 

These experiments were performed in a wider concentration regime to determine when saturation of the binding sites would occur, providing a more accurate fit for the calculation of the IFs.

### 2.4. Batch Rebinding Experiments Evaluated by HPLC

Rebinding experiments were conducted according to the same protocol described in Section 2.3., which includes mixing the corresponding MIP or NIP powder (10 mg) with PBS solutions (5 mL) of known concentrations of SSRIs or caffeine. After placing those suspensions on an orbital shaker for 90 min, the samples were passed through a Fisherbrand PTFE 0.45 µm filter membrane (Fisher Scientific, Loughborough, UK). The resulting solutions were analyzed with an Agilent 1100 Series HPLC equipped with a G1315B diode array detector and a Quat Pump G1311A (Agilent Technologies, Waldbronn, Germany) equipped with a 100-place auto-injector and diode-array UV absorbance detector (DAD) (220 nm). Caffeine is an important anthropogenic marker in surface water, and its levels can be directly related to water quality [23]. The chemical structure is significantly different from the studied SSRIs and therefore, it was used in experiments to study the selectivity, since it is important that the developed analytical tool can discriminate between various classes of micropollutants. 

Data analysis was carried out using ChemStation for LC software (version 10.02, Agilent Technologies, Wokingham, UK). Measurements were performed in triplicate in order to determine the standard deviation of the signal. Two sets of experimental parameters were used, one for the analysis of the individual antidepressants and one for the mixture of them. Both the initial (*C_i_*) and the free (*C_f_*) concentration were analyzed for binding. 

For individual analysis, a Hypersil ODS-2 C18 HPLC column (5 µm particle size, 150 × 4.6 mm, Thermo Fisher Scientific, Waltham, MA, USA) was used with a flow rate of 1.1 mL/min. The mobile phase was a 75:25 (*v*/*v*) mixture of acetonitrile and orthophosphoric acid solution (0.1% H_3_PO_4_ (aq), pH 2.1), with the injection volume set at 10 µL. For the mixed samples, a longer column (Gemini C18 HPLC column, 5 µm particle size, 250 × 4.6 mm, Phenomenex, Inc., Macclesfield, UK) was used along with sodium acetate buffer (pH 4.5) in the mobile phase to facilitate discrimination between the various drugs. The mobile phase was composed of the buffer along with acetonitrile and methanol (6.5:3.2:0.3), which was run at a flow rate of 1.1 mL/min using an injection volume of 20 µL.

## 3. Results and Discussion

### 3.1. Synthesis and Characterisation of Fluoxetine MIPs and NIPs

MIPs-1–7 and NIPs-1–7 polymers were synthesized in quantitative yields and analyzed via TGA. Thermogravimetric analysis analysis showed that the polymers were stable up until temperatures of 200 °C (Supplementary Figure S1). Rebinding experiments with MIP-1 and NIP-1 were performed to determine the influence of the time on binding of fluoxetine, with measurements taken at 15, 30, 60, 90, 120, 240, and 1020 min. The optimal binding time was seen as the shortest time at which high specific binding of fluoxetine was achieved. It was found that there was no significant increase in specific binding after 90 min, and therefore the binding time was fixed to this time in further experiments. The pH was kept neutral, as no significant difference between binding at pH 5 and 7 was observed, which meant adjustments of the PBS buffer were unnecessary (see Supplementary Figure S2). The size and morphology of the resulting particles was characterized by scanning electron microscopy (SEM) (see Supplementary Figure S3), with the size distribution ranging from approximately 1 to 25 µm (with some agglomeration during the preparation of the SEM sample).

### 3.2. Monomer Optimization Study 

Batch rebinding experiments were performed with PBS solutions (pH 7) containing fluoxetine concentrations ranging from 0 to 1 mM using UV–Vis spectrophotometry as the read-out technique. Binding isotherms were constructed by using both results obtained by UV–Vis and HPLC analysis. The binding of fluoxetine to the MIP and NIP layers is provided in Table 2. Imprint factors were calculated at two different concentrations for accuracy. An allometric fit was used to describe the MIP data, with all *R*^2^ values above 0.9. The data obtained for NIPs was best described with a linear correlation due to the nonspecific nature of the fluoxetine binding to the NIP layer. 

**Figure 2:**
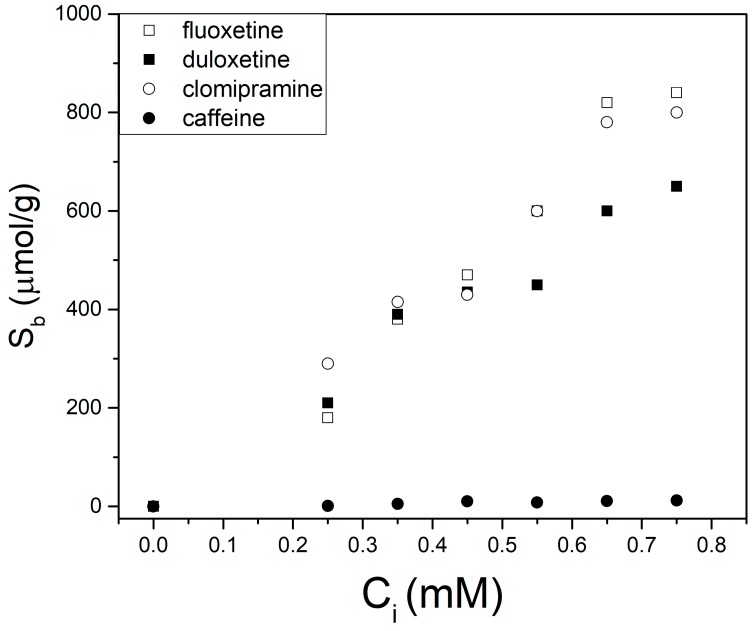
Binding isotherms of MIP-2 for the binding of fluoxetine (open squares), duloxetine (solid squares), clomipramine (open circles) and caffeine (solid circles).

MIP-1–3, synthesized with methacrylic-based monomers (MAA, IA, and HEMA, respectively; Table 1) exhibited a high amount of specific binding to fluoxetine (Table 2). The highest IF was achieved for MIP-2, probably due to higher charge density of the IA monomer, provided with two carboxylic acid groups [24]. MIP-4 and MIP-5, prepared using acrylamide and acrylonitrile, respectively, as the functional monomers, did not show any specificity towards the template, probably due to weak interactions between the amide and cyano group in the monomer and the amine on fluoxetine. MIP-6 and MIP-7 (Table 1) were prepared to evaluate the possible synergistic effect of having two functionalized monomers MAA/IA and MAA/HEMA, respectively. From the results (Table 2), we can see that MIP-6 has a very large IF but suffers from nonspecificity, as NIP-6 has a high degree of binding. MIP-7, on the other hand, does not benefit from the monomer mixture and shows low specificity. Based on the results, MIP-2, whose corresponding NIP-2 showed the lowest binding (Figure 2), was further evaluated for its recognition capability towards fluoxetine, duloxetine, clomipramine, and caffeine (Figure 1), using HPLC analysis. 

Caffeine is substantially different in structure from the other compounds, and thus shows minimal binding to MIP-2. This is clear from Figure 2, where binding does not exceed 20 µmol/g for caffeine whereas the other compounds all bind more than 600 µmol/g. Binding of fluoxetine at *C_i_* = 0.75 was equal to ≈800 µmol/g, similar to the results obtained to clomipramine. The fact that clomipramine (bearing a tertiary amine) behaves the same as fluoxetine (provided with a secondary amine) suggests that binding may not only be caused by H bonding but also by other noncovalent interactions. The binding of duloxetine at lower initial concentrations is similar to both these two compounds, but the overall binding capacity of ≈600 µmol/g is slightly lower. The reason for this could be that these compounds share a similar pharmacophore, and the main interaction of the functional monomer is with the nitrogen. 

### 3.3. HPLC Analysis of SSRI Mixtures

The viability of the method to separate mixtures from compounds of the same drug family was first evaluated by measuring a mixture with 1:1 ratio with each of the other compounds (*C_i_* = 0.75 mM). MIP-2 was used in these experiments, with fluoxetine as the template. Since HPLC is able to separate out this mixture, it can be determined whether binding to the polymers is a competitive process and what level of selectivity can be achieved. The binding time was kept at 1 h but the amount of the polymer was reduced from 10 to 5 mg due to the higher injection volume used in these measurements. This prevented saturation of the signal, and measured peak areas would fall within the calibration range. The retention time for all compounds is provided in Supplementary Table S1 and chromatograms are included in Supplementary Figures S4–S7. All compounds were present in an equimolar ratio.

Measurements were performed in a 0–0.75 mM concentration range in PBS. In each experiment, fluoxetine was present in a 1:1 ratio with another compound of the same family (duloxetine, and clomipramine) as well as structurally different drugs (caffeine) which allows competition for binding to occur. These mixtures are more representative for real samples, which will be composed of a variety of compounds present in different ratios. The differences in binding at a concentration of 0.25 mM between MIP-2 and NIP-2 for fluoxetine in the presence of other compounds are listed in Table 3. In addition, the binding of two other SSRIs and caffeine when mixed with fluoxetine is compared between MIP-2 and NIP-2 at a concentration of 0.25 mM. 

As expected, there is no difference in binding for MIP and NIP towards caffeine, as its structure is significantly different from fluoxetine. Fluoxetine had the highest specific binding compared to the other compounds, which shows that there is still an increased preference for the imprinted molecule even when there are substantial similarities in structure between the competing species. However, the imprint factors are significant lower compared to Table 2. This suggests that competition is indeed occurring and compounds within the same drug family can be bound to some degree with a single template. 

### 3.4. Dual Imprinted Polymer with Fluoxetine and Duloxetine

**Figure 3:**
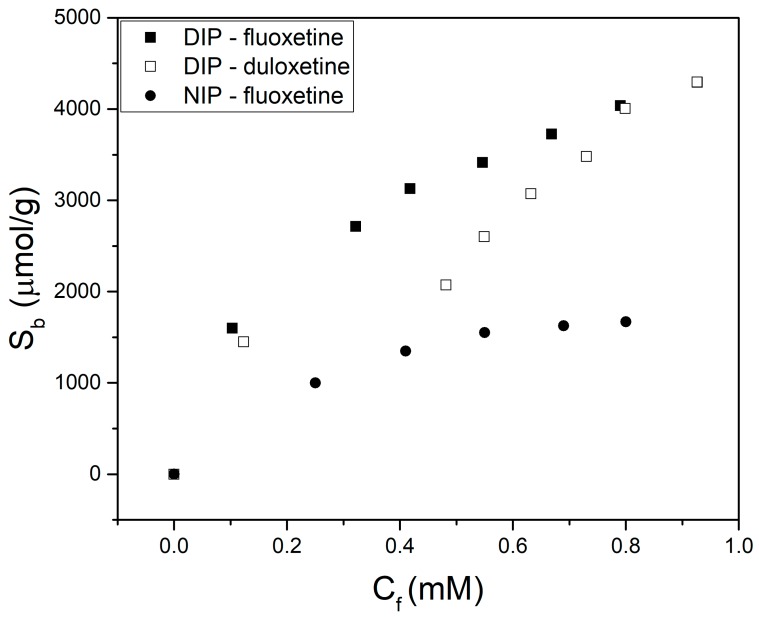
Dose–response curves of MIP-8 for fluoxetine (solid squares) and duloxetine (open squares), as well as for a nonimprinted polymer with fluoxetine (solid circles).

The presence of multiple templates on the MIPs’ performance was explored using fluoxetine and duloxetine. MIP-8 was prepared using IA as monomer and an equimolar mixture of fluoxetine and duloxetine as template. Batch rebinding experiments were performed on the resulting polymer MIP-8 (along with the nonimprinted corresponding analogue NIP-8) as previously outlined, with a concentration range from 0 to 2.5 mM for fluoxetine and duloxetine each (only fluoxetine was studied for NIP-8) in PBS. The results analyzed by HPLC are shown in Figure 3.

Both targets were successfully bound by the polymer, with increased levels seen at each concentration relative to the NIP. This demonstrates that both molecules can be imprinted simultaneously without inhibiting the polymer’s ability to rebind. Interestingly, more of the fluoxetine compared to the duloxetine was bound at lower concentrations, indicating the developed polymers had a preference for one target molecule. At higher concentrations, where binding sites are increasingly occupied, the binding of both target molecules converged. This indicates there is no significant difference between binding capacities of these polymers for the two target molecules, but there is a difference in the affinity of binding sites for fluoxetine and duloxetine. A larger library of these materials should be prepared in order to gain further insight in the properties of these dual-imprinted polymers. 

## 4. Conclusions

Imprinted polymers were successfully synthesized and optimized for fluoxetine, with various functional monomers investigated. The resulting material showed strong binding of the target molecule as well as compounds within the same drug family. Analysis using HPLC was able to show preferential binding of the target to the MIP for solutions containing multiple SSRIs although evidence of competition was present. These results indicate that high selectivity for SSRIs can be obtained using low-cost, easily synthesized imprinted polymers. Experimental parameters, such as concentration ratios and absorption time, can be adjusted in the future to tune the MIP’s selectivity further.

## Figures and Tables

**Table 1:** Monomers used for the synthesis of fluoxetine MIPs-1–7.

Molecularly Imprinted Polymer	Monomer
MIP-1	MAA
MIP-2	IA
MIP-3	HEMA
MIP-4	Acrylamide
MIP-5	Acrylonitrile
MIP-6	MAA + IA (1:1)
MIP-7	MAA + HEMA (1:1)

HEMA: 2-(Hydroxyethyl)methacrylate; IA: Itaconic acid; MMA: Methacrylic acid.

**Table 2:** Fluoxetine binding of MIPs and NIPs with various compositions as determined by UV–Vis spectroscopy.

Polymer	Binding at *C_f_* = 0.05 mM	Imprint Factor (*C_f_* = 0.05 mM)	Binding at *C_f_* = 0.2 mM (*S_b_*/*C_f_*)	Imprint Factor (*C_f_* = 0.2 mM)
MIP-1	1240	2.1	1250	2.2
NIP-1	600		575	
MIP-2	900	5.6	850	6.3
NIP-2	160		135	
MIP-3	1320	2.8	1300	2.8
NIP-3	480		465	
MIP-4	360	2.6	370	2.6
NIP-4	140		145	
MIP-5	300	1.1	290	1.1
NIP-5	260		255	
MIP-6	3600	7.2	3555	7.2
NIP-6	500		495	
MIP-7	520	2.0	525	1.5
NIP-7	320		340	

**Table 3:** Imprint factors for MIP-2 and NIP-2 of fluoxetine in solutions containing both fluoxetine and other drug compounds at 0.25 mM.

Compounds Mixture	IF for Fluoxetine	MIP/NIP Comparison Compounds
Fluoxetine:Duloxetine	1.45	1.26
Fluoxetine:Clomipramine	1.58	1.37
Fluoxetine:Caffeine	2.19	0.71

## Supplementary Materials

The following are available online at https://www.mdpi.com/2313-7673/4/1/18/s1, Figure S1: Thermogravimetric analysis curves for MIP-1, -2, -3, -6, and -7, Figure S2: Influence of binding time and pH on binding of the template to MIPs, Figure S3: Size and morphology of particles, Figure S4: Chromatogram for fluoxetine, Figure S5: Chromatogram for duloxetine, Figure S6: Chromatogram for clomipramine, Figure S7: Chromatogram for caffeine, Figure S8: FTIR spectrum of MIP-2 imprinted with fluoxetine, Table S1: HPLC retention times of the compounds fluoxetine, duloxetine, clomipramine, and caffeine.
