# Supplementary material for: Synthesis of Optimized Molecularly Imprinted Polymers for the Isolation and Detection of Antidepressants via HPLC
Source: Biomimetics (Basel). 2019 Feb 20;4(1):18. doi: 10.3390/biomimetics4010018 (PMC6477609; doi:10.3390/biomimetics4010018)
Supplement: Supplementary file 1 [file biomimetics-04-00018-s001.pdf]

Biomimetics **2019**, *4*, 18

Synthesis of Optimized Molecularly Imprinted Polymers for the Isolation and Detection of Antidepressants via HPLC †

Alexander D. Hudson [1,*] Ricard Sola Mestres [1], Jorge T. Ueta [2], William Battell [1,3], Oliver Jamieson [1], Thomas Dunbar [1], Beatriz Maciá [1] and Marloes Peeters [1]

[1] Manchester Metropolitan University, Faculty of Science and Engineering, Division of Chemistry and Environmental Science, Manchester M1 5GD, UK; rsolamestres@gmail.com (R.S.M.); oliver.jamieson@stu.mmu.ac.uk (O.J.); thomasdunbar1993@gmail.com (T.D.); B.Macia-Ruiz@mmu.ac.uk (B.M.); m.peeters@mmu.ac.uk (M.P.)

[2] Departamento de Engenharia Química, Escola Politécnica, Universidade de São Paulo, Avenida Prof. Luciano Gualberto, Travessa 3, 380, CEP05508-900 São Paulo, Brazil; tadinchnails@gmail.com

[3] Department of Chemical Engineering, Centre for Advanced Separations Engineering, University of Bath, Claverton Down, Bath BA2 7AY, UK; W.Battell@bath.ac.uk

* Correspondence: a.hudson@mmu.ac.uk; Tel.: +44-161-247-1450

† This article is an extended version of our paper presented at the Bioinspired Materials 2018 conference; paper No. P3.

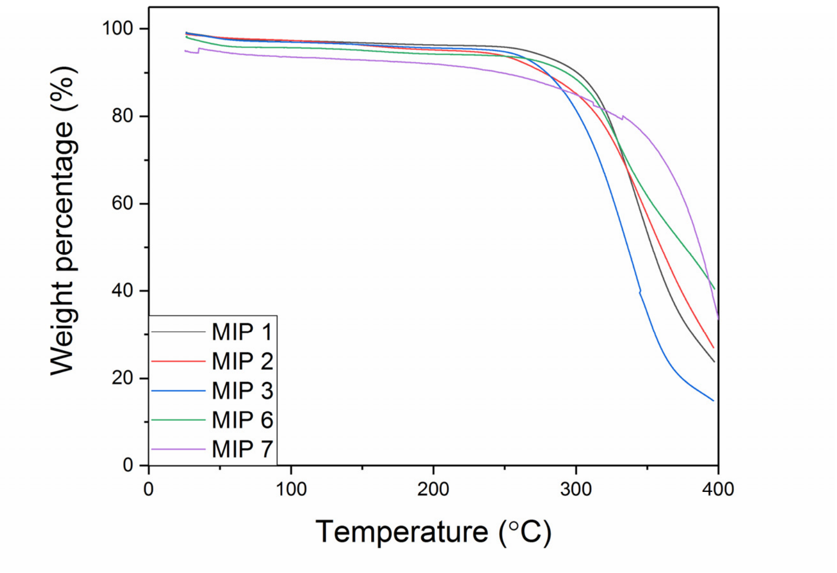

Figure S1. Thermogravimetric analysis curves for MIP-1, -2, -3, -6, and -7. No noticeable weight loss was observed until ≈250 °C, demonstrating the stability of the polymer particles at elevated temperatures.

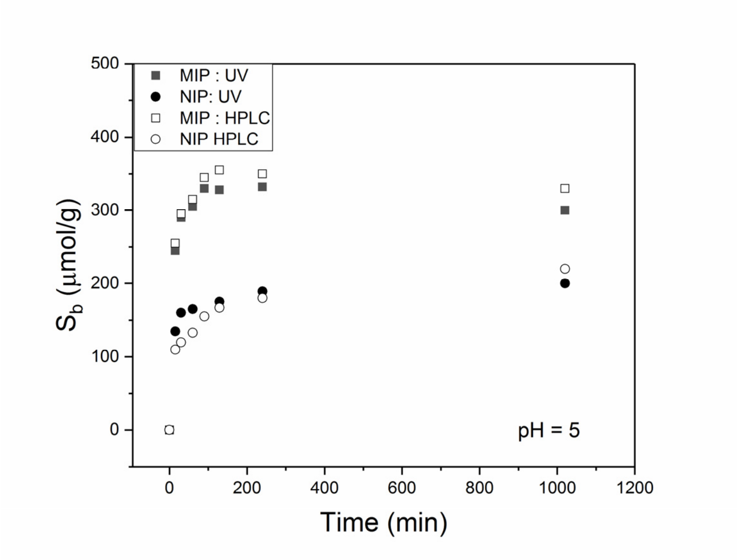

(a)

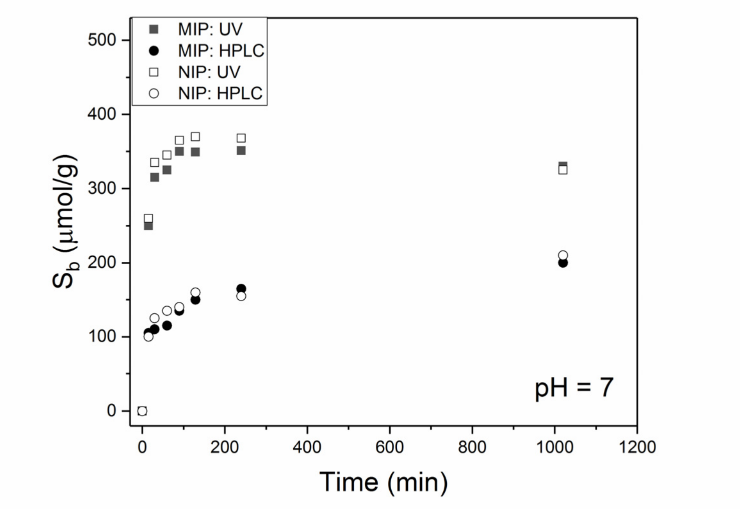

(b)

Figure S2. Influence of binding time and pH on binding of the template to MIPs. The effect of the time (15–1020 min) on binding of fluoxetine to the polymers was evaluated with MIP-1 and NIP-1. The results of two independent experiments, performed in PBS of (**a**) pH 5 and (**b**) 7 and with a fluoxetine concentration of C_i = 0.5 mM, were analyzed by UV–Vis (squares) and HPLC (circles).

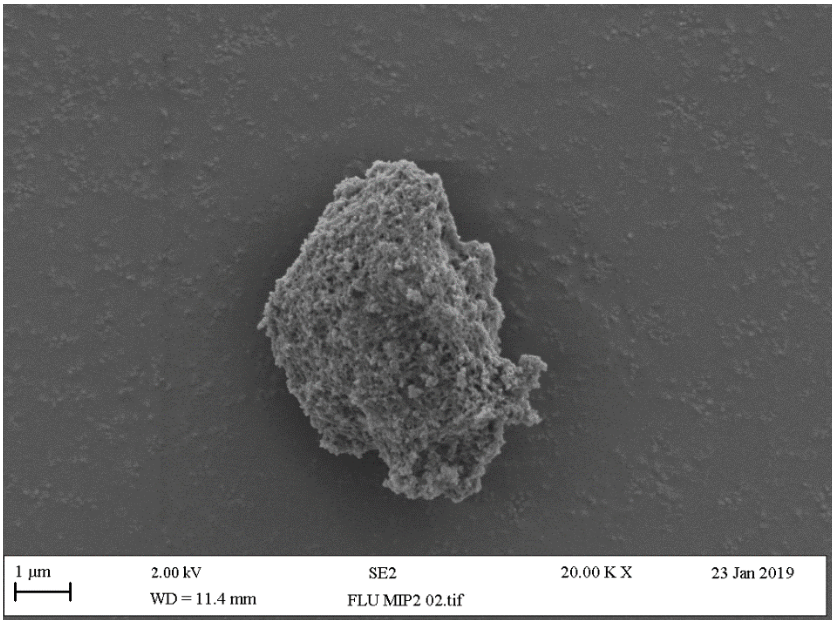

(a)

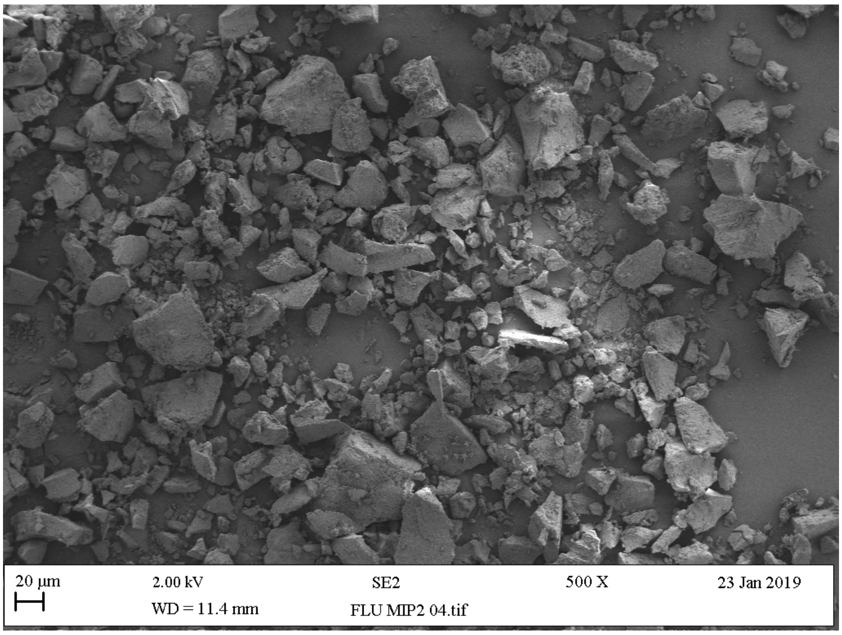

(b)

Figure S3. Size and morphology of particles. (**a**) Scanning electron micrograph of a particle produced from MIP-2 with fluoxetine as the template and (**b**) particle size distribution (below).

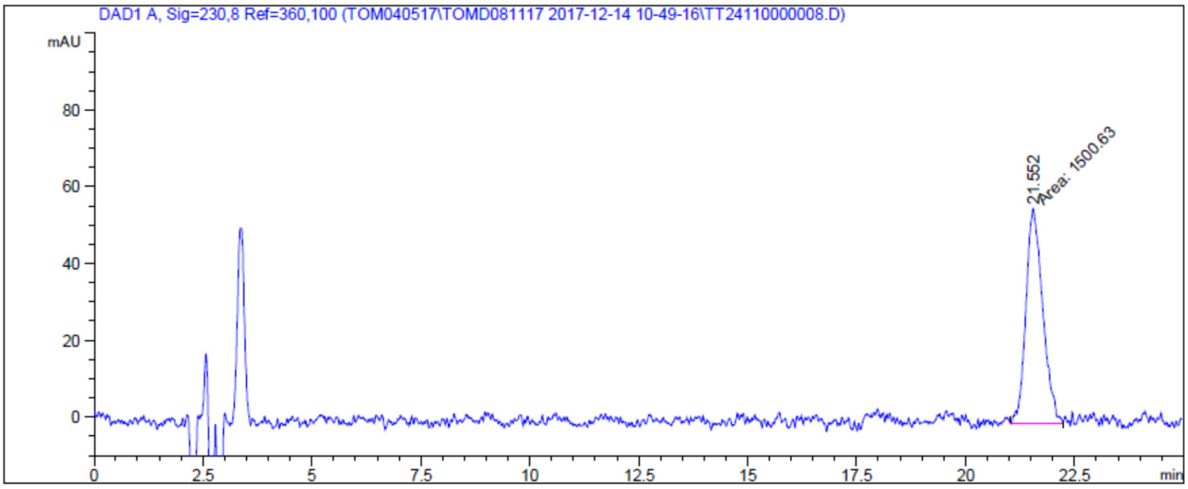

(a)

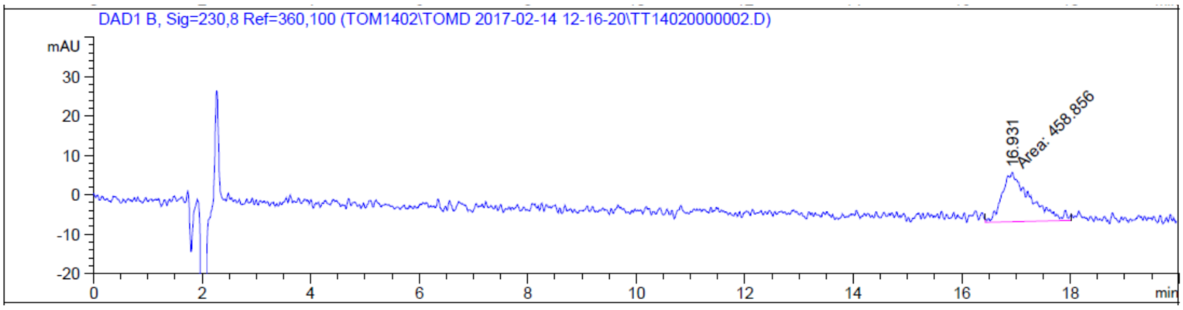

(b)

Figure S4. Chromatogram for fluoxetine. (**a**) Gemini C18 HPLC column, 5 µm particle size, 250 × 4.6 mm (Phenomenex, Inc.) in sodium acetate buffer (pH 4.5) with acetonitrile and methanol (6.5:3.2:0.3) at a flow rate of 1.1 mL/min. (**b**) Hypersil ODS-2 C18 HPLC column, 5 µm particle size, 150 × 4.6 mm (Thermo Fisher Scientific) in acetonitrile:orthophosphoric acid solution (0.1% H_3PO_4 (aq), pH 2.1), 75:25 (*v*/*v*) at a flow rate of 1.1 mL/min.

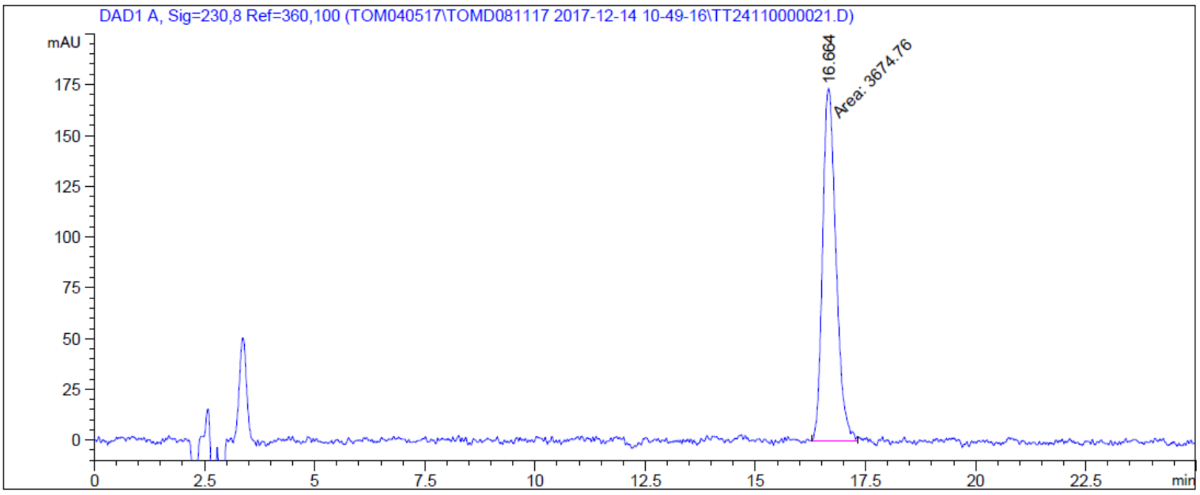

(a)

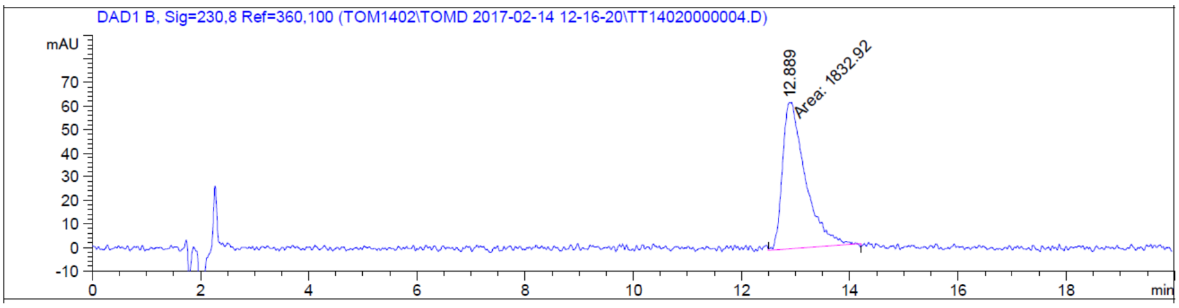

(b)

Figure S5. Chromatograms for duloxetine. (**a**) Gemini C18 HPLC column, 5 µm particle size, 250 × 4.6 mm (Phenomenex, Inc.) in sodium acetate buffer (pH 4.5) with acetonitrile and methanol (6.5:3.2:0.3) at a flow rate of 1.1 mL/min. (**b**) Hypersil ODS-2 C18 HPLC column, 5 µm particle size, 150 × 4.6 mm (Thermo Fisher Scientific) inacetonitrile:orthophosphoric acid solution (0.1% H_3PO_4 (aq), pH 2.1), 75:25 (*v*/*v*) at a flow rate of 1.1 mL/min.

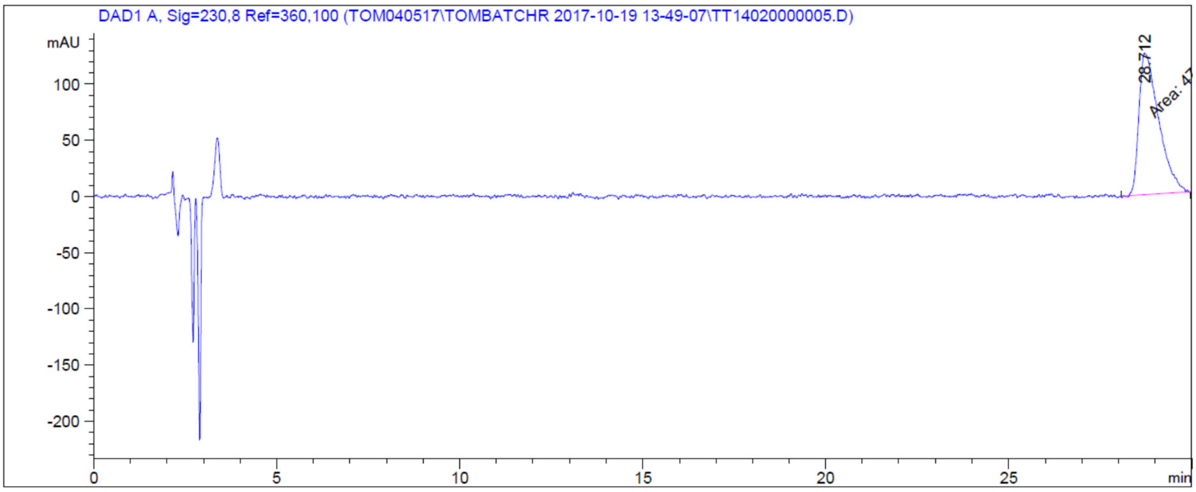

Figure S6. Chromatogram for clomipramine. Gemini C18 HPLC column, 5 μm particle size, 250 × 4.6 mm (Phenomenex, Inc.) in sodium acetate buffer (pH 4.5) with acetonitrile and methanol (6.5:3.2:0.3) at a flow rate of 1.1 mL/min.

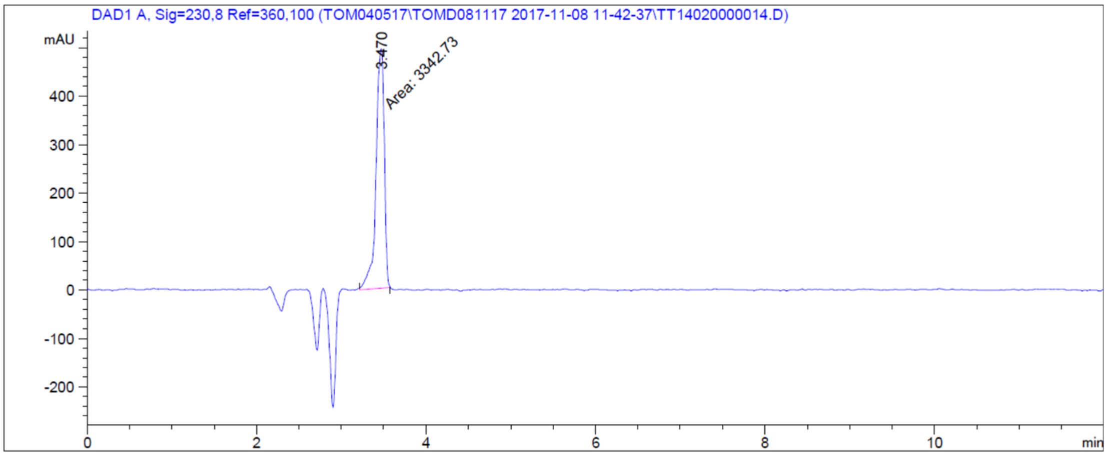

Figure 7. Chromatogram for caffeine. Gemini C18 HPLC column, 5 μm particle size, 250 × 4.6 mm (Phenomenex, Inc.) in sodium acetate buffer (pH 4.5) with acetonitrile and methanol (6.5:3.2:0.3) at a flow rate of 1.1 mL/min.

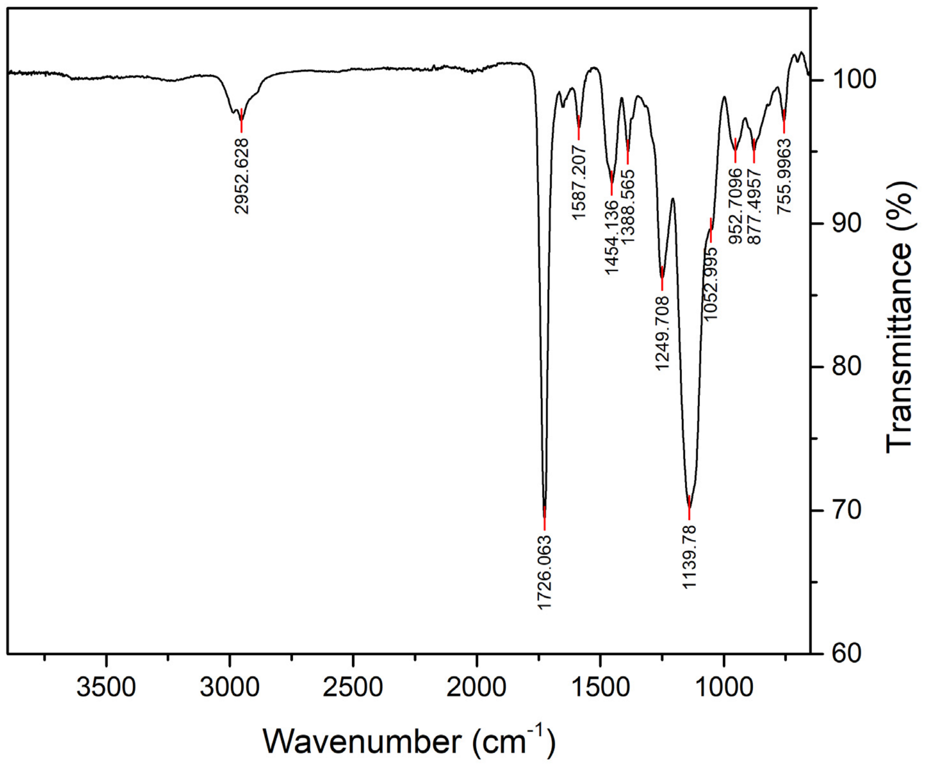

Figure S8. FTIR spectrum of MIP-2 imprinted with fluoxetine. Spectrum shows the typical polymer peaks at 3000 cm^{-1} for the C–H stretch and the C=O functionality with a peak around 1700 cm^{-1}.

Table S1. HPLC retention times of the compounds fluoxetine, duloxetine, clomipramine, and caffeine.

**Compound**	**Retention time (min)** [1]
Fluoxetine	21.7
Duloxetine	16.7
Clomipramine	29.0
Caffeine	3.6

[1] Gemini C18 HPLC column, 5 µm particle size, 250 × 4.6 mm (Phenomenex, Inc.) in sodium acetate buffer (pH 4.5) with acetonitrile and methanol (6.5:3.2:0.3) at a flow rate of 1.1 mL/min.
